# The extracellular fluid macromolecular composition differentially affects cell-substrate adhesion and cell morphology

**DOI:** 10.1038/s41598-019-44960-3

**Published:** 2019-06-11

**Authors:** Jordi Gonzalez-Molina, Joana Mendonça da Silva, Barry Fuller, Clare Selden

**Affiliations:** 1 UCL Institute for Liver and Digestive Health, Royal Free Hospital Campus, UCL Medical School, University College London, NW3 2PF London, UK; 2 Microbiology, Tumor and Cell Biology Department, Karolinska Institutet, 171 65 Solna, Sweden; 3 Oncology-Pathology Department, Karolinska Instituet, 171 76 Stockholm, Sweden; 4 Department of Surgical Biotechnology, Royal Free Hospital, UCL Medical School, University College London, NW3 2QG London, UK

**Keywords:** Biophysics, Biomaterials - cells, Cancer microenvironment, Mechanotransduction

## Abstract

Soluble macromolecules present in the tumour microenvironment (TME) alter the physical characteristics of the extracellular fluid and can affect cancer cell behaviour. A fundamental step in cancer progression is the formation of a new vascular network which may originate from both pre-existing normal endothelium and cancer-derived cells. To study the role of extracellular macromolecules in the TME affecting endothelial cells we exposed normal and cancer-derived endothelial cells to inert polymer solutions with different physicochemical characteristics. The cancer cell line SK-HEP-1, but not normal human umbilical vein endothelial cells, responded to high-macromolecular-content solutions by elongating and aligning with other cells, an effect that was molecular weight-dependent. Moreover, we found that neither bulk viscosity, osmotic pressure, nor the fractional volume occupancy of polymers alone account for the induction of these effects. Furthermore, these morphological changes were accompanied by an increased extracellular matrix deposition. Conversely, cell-substrate adhesion was enhanced by polymers increasing the bulk viscosity of the culture medium independently of polymer molecular weight. These results show that the complex macromolecular composition of the extracellular fluid strongly influences cancer-derived endothelial cell behaviour, which may be crucial to understanding the role of the TME in cancer progression.

## Introduction

Cancer cells require a constant supply of nutrients, oxygen, and the removal of waste products. Thus, the formation of a new vascular network, a process termed angiogenesis, is a pivotal step in tumour growth and progression^1,2^. Moreover, angiogenesis also facilitates the penetration and circulation of cancer cells into the vascular network, which allows the spreading of cancer cells to adjacent and distant organs, a process known as metastasis. The formation of new vessels can originate from endothelial cells of pre-existing vessels, from newly recruited cells from the bone marrow, and can also derive from the angiogenic differentiation of tumour stem cells^3,4^. To promote angiogenesis within tumours, cancer cells adapt the tumour microenvironment (TME) so as to acquire pro-angiogenic features. Cancer cells, and stromal cells influenced by the presence of cancer cells, are known to modify biophysical characteristics of the TME such as the stiffness and architecture of the extracellular matrix (ECM) by secreting ECM proteins and ECM-remodelling enzymes. These changes in the ECM have been reported to affect endothelial cell morphology and vascular permeability which can facilitate cancer cell intra- and extra-vasation^5,6^. Endothelial cells also respond to haemodynamic forces including cyclic strain and shear stress. These biophysical signals have major effects on the structural organisation of the cytoskeleton, cell-cell junctions, and the adhesion of cells to their supporting ECM through mechano-sensitive structures such as focal adhesions, ion channels, and the junctional mechanosensory complex^7^. The downstream signals of these structures converge in the regulation of the actin cytoskeleton through proteins involved in actin remodelling such as Rho GTPases^8–10^. For instance, fluid shear stress induces cell alignment in the direction of the flow through a process mediated by the GTPases RhoA and Rac1^10^.

Cancer cells not only modify the solid component of the TME but are also constantly secreting soluble macromolecules into the extracellular fluid. The secretion of these macromolecules changes the physicochemical properties of the extracellular fluid, such as the osmotic pressure, and induces the macromolecular crowding effect. Macromolecular crowding is a biophysical phenomenon causing both the excluded volume effect, which enhances reaction rates due to the increased effective concentration of the molecules involved in a reaction, and conversely, the enhanced effective viscosity, which reduces diffusion and, thus, reaction rates^11^. These effects depend on the molecular concentration and size of the macromolecules as well as the physicochemical characteristics of the molecules involved in the reaction^11–13^. Cells secrete macromolecules with a vast range of characteristics, for instance, some adenocarcinoma cells secrete large viscosity-enhancing macromolecules such as mucus-forming mucins, which have been linked to poor disease outcome^14^. The presence of macromolecules enhances ECM deposition and reaction rates at the cell surface^15,16^. Moreover, high viscosity-inducing macromolecules affect cell-ECM adhesion dynamics in cancer cells^17^.

Thus, we hypothesised that the macromolecular content of the TME affects the response of endothelial cells to their microenvironment and sought to characterise the properties of macromolecules inducing these effects. To this end, we used polymers of varying type, concentration, and molecular weight to modify the macromolecular content of the microenvironment of a liver metastasis endothelial-derived cell line, SK-HEP-1 and non-cancerous human umbilical vein endothelial cells (HUVEC). We found that SK-HEP-1 but not HUVEC cells present enhanced cell spreading area, elongation, and multicellular alignment upon exposure to polymer-containing solutions. These morphological changes were found to be independent of actin cytoskeleton integrity. Finally, we found that these effects are polymer molecular weight and type-dependent and, contrary to the effects on cell-substrate adhesion, are bulk viscosity-independent. Altogether, these results indicate that the macromolecular content of the TME has major effects on endothelial-derived cells, which may have consequences on cancer progression.

## Results

### Normal and cancerous endothelial-derived cells respond to Na-alginate differently

**Figure 1:**
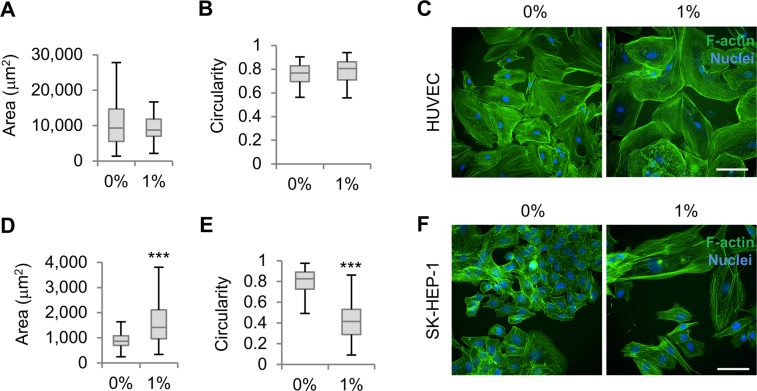
Na-alginate induces morphological changes in SK-HEP-1 cells but not in HUVECs. (**A**,**B**) Cell area (**A**) and circularity (**B**) of HUVECs exposed to control (0%) or 1% Na-alginate-containing medium (1%) (n > 57 cells). (**C**) Fluorescence images of HUVECs after 0% or 1% Na-alginate treatment. Scale bar, 50 μm. (**D**,**E**) Quantification of cell area (**D**) and circularity (**C**) of SK-HEP-1 cells treated for 4 days with 0% or 1% Na-alginate-containing medium (n = 250 cells). (**F**) Fluorescence images of SK-HEP-1 cells after treatment with 0% or 1% Na-alginate. Scale bar, 50 μm. Boxplots represent the median, first, and third quartiles; whiskers indicate maximum and minimum within 1.5x the interquartile range. Statistical significance was assessed by two-tailed Student’s *t*-test. ***p < 0.001.

To investigate the effect of extracellular macromolecules on endothelial cell behaviour we first compared the responses to polymeric solutions of normal human umbilical vein endothelial cells (HUVEC) and SK-HEP-1 cells, a liver metastasis cell line of endothelial origin. HUVECs have long been used in studies of shear stress, showing rapid morphological changes and cell alignment under fluid shear stress conditions^18,19^. SK-HEP-1 cells have been used as a model of a cancer-derived endothelial-like cell^20^ and demonstrate enhanced cell migration upon exposure to viscous polymeric solutions^17^. First, we chose high molecular weight (250,000–320,000 g mol^−1^) and high viscosity sodium alginate (Na-alginate)^21^, an inert polymer extensively used as a biomaterial for cell therapy and tissue engineering^22^, since any effects found on endothelial cells may have repercussions on its biomedical applications. Furthermore, the molecular weight and viscosity of Na-alginate are within the range of those of mucins and mucinous fluids^23^, making this polymer also useful to mimic mucinous microenvironments found in mucin-producing tumours. Exposing HUVECs for up to 4 days to 1% Na-alginate, a solution with a viscosity of 47.6 cP (100 s^−1^ shear rate) which is considerably higher than that of cell culture medium (∼1 cP), did not induce major changes in HUVEC morphology, with cells maintaining their original area and circularity (Fig. 1A,B). Moreover, cell alignment was not observed, and the direction of actin stress fibres appeared to be isotropic (Supplementary Fig. S1 and Fig. 1C). Conversely, exposing SK-HEP-1 cells to 1% Na-alginate for 4 days resulted in cell elongation and a larger cell area than control cells (Fig. 1D,E). However, cell alignment could not be assessed as SK-HEP-1 cells exposed to 1% Na-alginate appeared scattered, an effect that could be a result of a reduced proliferation and enhanced migration^17^. These data show that cells of endothelial origin respond to high macromolecular-content environments and indicate that cancer-derived and normal endothelial cells respond differently to them. Interestingly, these effects do not occur rapidly (i.e. on a time scale of hours) as seen in fluid shear stress-induced effects but are only observed after 3–4 days of exposure, suggesting that the mechanisms regulating SK-HEP-1 responses to 1% Na-alginate may not be the same as in fluid shear-stress.

### SK-HEP-1 cells adhered to compliant substrates respond rapidly to Na-alginate

**Figure 2:**
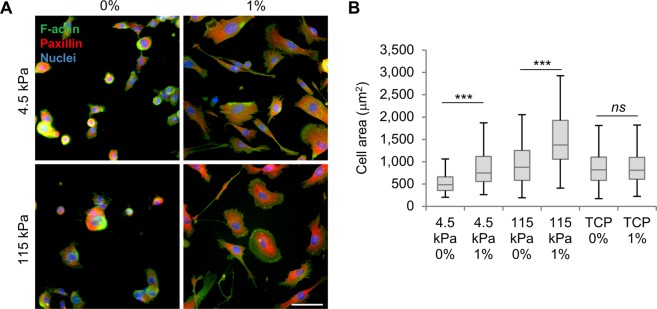
Na-alginate enhances SK-HEP-1 cell area on compliant substrates after 24 h. (**A**) Immunofluorescence of SK-HEP-1 cells adhered to Col-PAA gels and exposed to control (0%) or 1% N-alginate (1%) for 24 h. (**B**) Quantification of cell area of cells from (**A**) or cells adhered to tissue culture polystyrene (TCP) (n ≥ 100 cells). Boxplots represent the median, first and third quartiles; whiskers indicate maximum and minimum within 1.5x the interquartile range. Statistical significance was assessed by two-tailed Student’s *t*-test. *ns* p > 0.05, ***p < 0.001.

Extracellular fluid viscosity has been shown to affect cell-substrate adhesion and mechanosensing^17^. Thus, we evaluated whether SK-HEP-1 cells responded to a 1% Na-alginate solution when adhered to compliant *in vivo*-like substrates. Collagen-functionalised polyacrylamide (Col-PAA) gels were used as compliant substrates with defined elastic modulus. Cells were seeded on 4.5 kPa Col-PAA gels, mimicking the liver TME^24^, and supra-physiologically stiff 115 kPa Col-PAA gels, which is considerably softer than tissue culture polystyrene (∼1 GPa). In line with previous reports^25^, cell area was significantly larger when cells were adhered to 115 kPa than on 4.5 kPa gels (Fig. 2). Interestingly, in both Col-PAA gel stiffness, exposing cells to 1% Na-alginate caused a rapid enhancement of the cell spreading area, which occurred after less than 24 h of exposure (Fig. 2B). These results indicate that, in TME-like substrates, SK-HEP-1 cells rapidly respond to a viscous 1% Na-alginate solution, effects that are not seen when cells are adhered to very stiff tissue culture polystyrene at this exposure time. These different behaviours could be due to both the different mechanical properties of the substrates and the biochemical characteristics of collagen-based adhesion in Col-PAA gels compared to tissue culture polystyrene substrates. This suggests that the effect of Na-alginate on substrate adhesion, which occurs rapidly and is mediated by integrin-containing focal adhesions^26^, may not explain the morphological effects observed on cells adhered to tissue culture polystyrene after 4 days (Fig. 1D–E). This could be because the long-term effects, causing cell elongation and larger cell area, involve changes other than direct cell-substrate adhesion.

### Polymer-containing microenvironments cause SK-HEP-1 cell alignment

**Figure 3:**
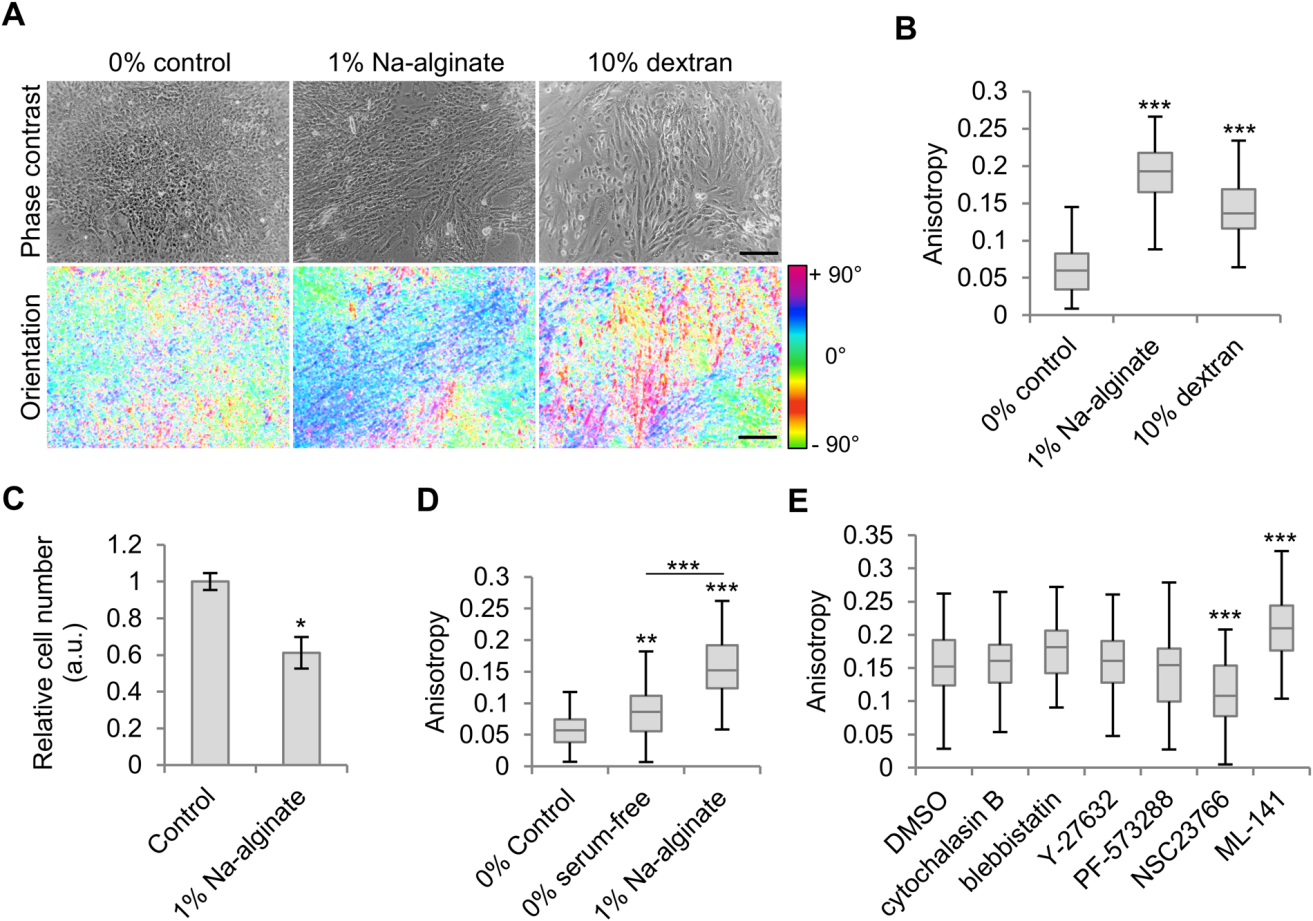
SK-HEP-1 cells align under viscous polymer conditions. (**A**) Phase contrast images of SK-HEP-1 cells after a 4-day polymer exposure and their corresponding pixel orientations pseudo-coloured according to their angle and saturation representing coherency. Scale bars, 200 μm. (**B**) Anisotropy quantification of SK-HEP-1 cell groups from (**A**) (n = 30 fields per condition). (**C**) SK-HEP-1 cells exposed to 1% Na-alginate for 4 days show a reduced cell number relative to control medium (n = 3). (**D**) Cells treated for 4 days with 0% Na-alginate serum-containing and serum-free media as well as serum-containing 1% Na-alginate medium were analysed for cell anisotropy (n = 60 fields). (**E**) Cell monolayer anisotropy of SK-HEP-1 cells exposed to 1% Na-alginate containing small molecule inhibitors or control (DMSO) for 4 days (n = 60 fields). Bar graphs indicate average ± s.e.m. Boxplots represent the median, first, and third quartiles; whiskers indicate maximum and minimum within 1.5x the interquartile range. Statistical significance was assessed by Student’s *t*-test (**C**) or one-way ANOVA with Tukey’s multiple comparison test. *p < 0.05, **p < 0.01, ***p < 0.001.

To evaluate whether SK-HEP-1 cell morphological changes result in cellular alignment upon exposure to high macromolecular-content microenvironments, as seen in shear stress-induced endothelial cells, 50% confluent cell cultures were exposed to 1% Na-alginate. Also, to avoid the effect of alginate precipitate caused by crosslinking alginate with calcium present in the culture medium or changes in cell-available calcium, 10% dextran solution (25.8 cP), which contrary to Na-alginate behaves as a Newtonian fluid, was also used^27^. After a 4-day exposure to polymer-containing solutions, an analysis of cell orientation revealed alignment of groups of cells in both alginate- and dextran-exposed cells, indicating that both Newtonian and non-Newtonian fluids can induce SK-HEP-1 cell alignment (Fig. 3A). However, compared to endothelial cells exposed to fluid flow^18,28,29^, not all cells oriented in the same direction but rather groups of aligned cells oriented in different directions. Cell alignment was further quantified by cell anisotropy based on image analysis of phase-contrast images, which revealed a significant enhancement of cell anisotropy in cells exposed to both 1% Na-alginate and 10% dextran (Fig. 3B). As SK-HEP-1 cell cultures exposed to Na-alginate were less confluent than control cells after a 4-day treatment (Fig. 1F), we investigated the effect of 1% Na-alginate on cell proliferation. Cell cultures exposed to 1% Na-alginate had a significantly lower cell number than control cultures after 4 days (Fig. 3C). Thus, the influence of a reduced cell proliferation on the quantification of cell alignment by cell anisotropy analysis was evaluated. SK-HEP-1 cells in serum-free polymer-free medium were used to study the effect of lower proliferation rates, due to starvation, on cell anisotropy, demonstrating an enhanced anisotropy in starved cells (Fig. 3D). However, cell anisotropy in starved cells was significantly lower than in 1% Na-alginate-exposed cells indicating that reduced cell density alone does not explain the cell anisotropy and alignment observed in polymer-treated SK-HEP-1 cells.

### Na-alginate-induced SK-HEP-1 cell alignment is affected by Rac1 and cdc42 inhibition but not actin disruption

Next, small molecule inhibitors were used to study the role of actomyosin, FAK, and Rho GTPases in cell alignment as these have been previously shown to be involved in endothelial cell alignment^8–10,30–32^. Unexpectedly, disruption of the actin cytoskeleton or inhibition of its contractility did not abrogate Na-alginate-induced cell anisotropy (Fig. 3E). To control that cytochalasin B-induced actin disruption in 1% Na-alginate media was effective, cells were stained for F-actin. Control cells presented visible actin stress fibres but cytochalasin B-treated cells exhibited a punctate staining (Supplementary Fig. S2A). Moreover, cytochalasin B inhibition also caused a size reduction of paxillin-containing focal adhesions compared to larger and elongated focal adhesions observed in control cells (Supplementary Fig. S2A). Furthermore, SK-HEP-1 cell monolayers treated with cytochalasin B exhibited a significantly lower actin fibre anisotropy compared to untreated cells indicating that the actin disruption was effective (Supplementary Fig. S2B,C). Similar to actin disruption, inhibition of FAK did not cause significant effects on cell anisotropy, a response that has also been observed in endothelial cell alignment caused by topographic features^33^. Conversely, inhibition of Rac1 and cdc42 decreased, and increased, cell anisotropy, respectively. These observations are in line with previous reports on shear stress-induced endothelial cell alignment^10^ and suggest that actin polymerisation mediated by Rho GTPases, but not its stabilisation, may be required for their role in cell alignment as actin depolymerisation did not abrogate cell alignment. These results show that SK-HEP-1 cells exposed to viscous polymer-containing solutions present responses that are reminiscent of shear stress-exposed endothelial cells, but the intracellular molecular players may differ and be, instead, more similar to topography-induced cell alignment^33,34^.

### Polymer molecular weight is crucial in determining SK-HEP-1 morphological and alignment responses

**Figure 4:**
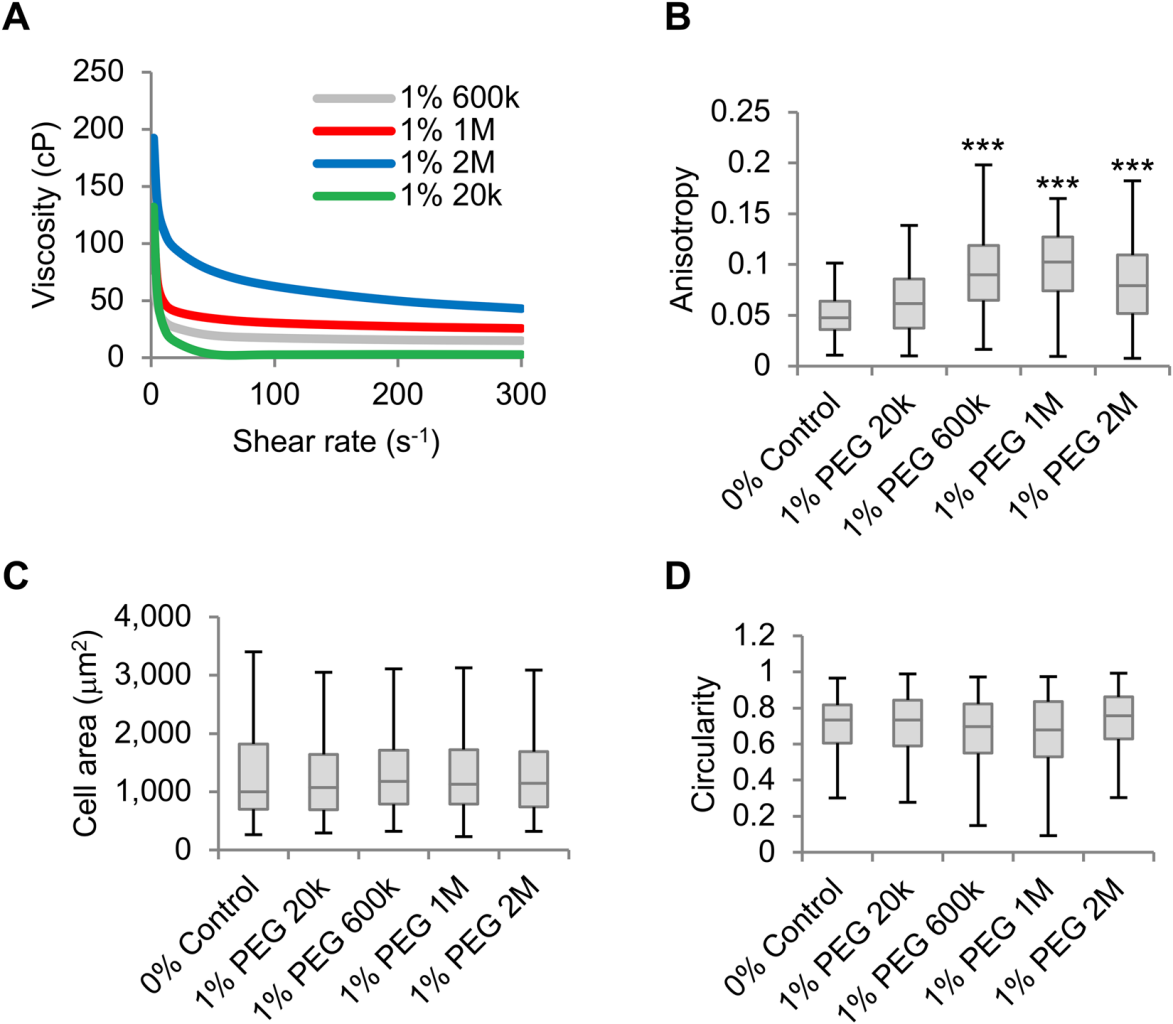
High viscosity PEGs do not reproduce the effects of Na-alginate on SK-HEP-1 cells. (**A**) Viscosity to shear rate relationship of PEG solutions with varying molecular weights. (**B**) Anisotropy of cell monolayers exposed for 4 days to PEG-containing media (n = 60 fields). (**C**,**D**) Cell area (**C**), and circularity (**D**) of cells from (**B**) (n ≥ 294 cells). Boxplots represent the median, first, and third quartiles; whiskers indicate maximum and minimum within 1.5x the interquartile range. Statistical significance was assessed by one-way ANOVA with Tukey’s multiple comparison test. ***p < 0.001.

**Figure 5:**
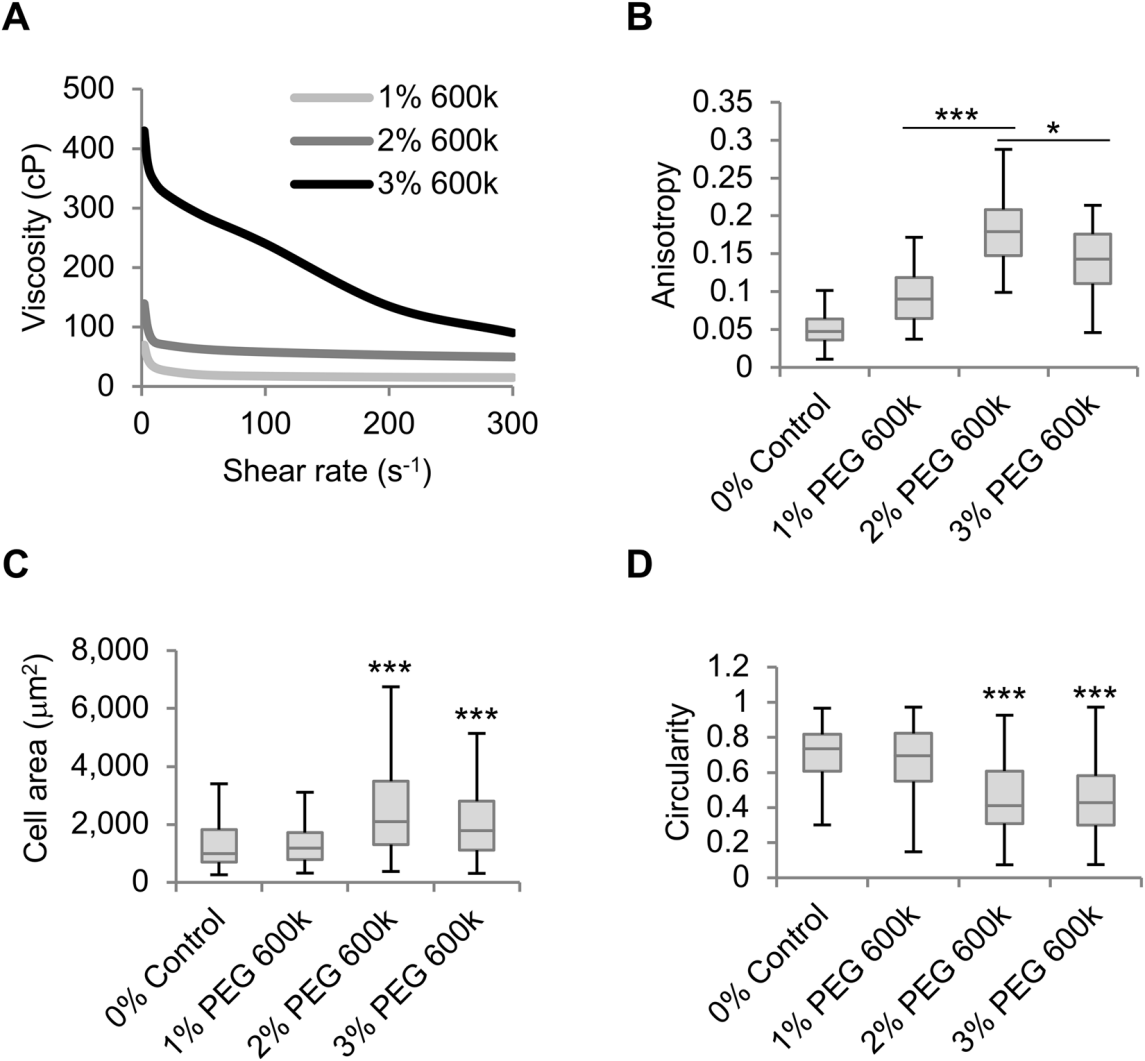
PEG 600,000 induces SK-HEP-1 cell alignment. (**A**) Viscosity to shear rate relationship of PEG 600,000 solutions at varying concentrations. (**B**) Anisotropy of cell monolayers exposed for 4 days to PEG 600,000 (PEG 600k) (n = 60 fields). (**C**,**D**) Cell area (**C**), and circularity (**D**) of cells from (**B**) (n ≥ 294 cells). Boxplots represent the median, first, and third quartiles; whiskers indicate maximum and minimum within 1.5x the interquartile range. Statistical significance was assessed by one-way ANOVA with Tukey’s multiple comparison test. *p < 0.05; ***p < 0.001.

Next, we sought to determine the molecular characteristics of polymers inducing SK-HEP-1 cell morphological changes and alignment. Initially, polyethylene glycol (PEG) polymers with various defined molecular weights were used at 1% w/v. The bulk viscosity of PEG solutions increased with molecular weight reaching 62.6 cP with PEG 2,000,000 g mol^−1^ (PEG 2M), a viscosity higher than 1% Na-alginate (Fig. 4A). 1% PEG solutions between 600,000–2,000,000 g mol^−1^ enhanced cell anisotropy only to levels comparable to serum-starved cells but not to 1% Na-alginate-exposed cells (Figs 3D and 4B). Furthermore, no 1% PEG solution, including PEG 2M, caused enhanced cell area and elongation as observed in 1% Na-alginate-treated cells (Fig. 4C,D). As 10% dextran with a molecular weight of 450,000–650,000 g mol^−1^ caused cell alignment, we speculated that PEG 600,000 (PEG 600k) at higher concentrations could also induce morphological effects on SK-HEP-1 cells. Increasing PEG 600k concentration dramatically enhanced the bulk viscosity of the solution (Fig. 5A). Interestingly, 2% PEG 600k, with a viscosity of 57.8 cP, significantly increased cell anisotropy and also caused cell elongation and a significantly enhanced cell area (Fig. 5B–D).

**Figure 6:**
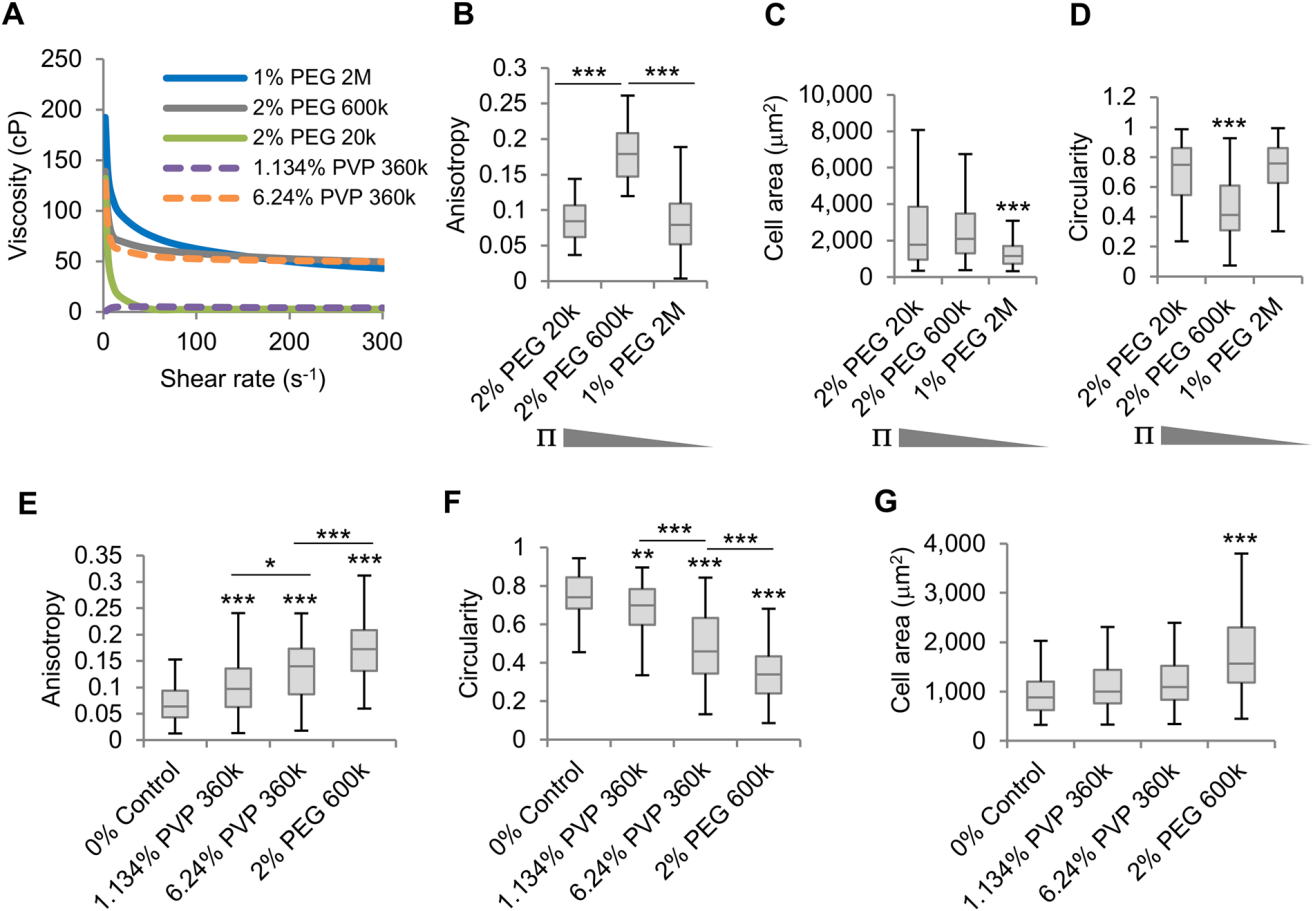
Bulk viscosity and macromolecular crowding alone do not cause cell alignment. (**A**) Viscosity to shear rate relationship of PEG solutions. (**B**) Anisotropy of cell monolayers exposed for 4 days to PEG solutions (n > 40 fields). (**C**,**D**), Cell area (**C**), and circularity (**D**) of cells from (**B**) (n ≥ 163 cells). ∏ represents the osmotic pressure. (**E**) Anisotropy of cell monolayers exposed for 4 days to PEG or PVP solutions (n = 80 fields). (**F**,**G**), Circularity (**C**), and cell area (**G**) of cells from (**E**) (n = 100 cells). Boxplots represent the median, first, and third quartiles; whiskers indicate maximum and minimum within 1.5x the interquartile range. Statistical significance was assessed by one-way ANOVA with Tukey’s multiple comparison test. ***p < 0.001.

Enhancing PEG 600k concentration has a consequent effect on osmotic pressure and crowding. Thus, to investigate whether osmotic pressure or macromolecular crowding are the main cause of SK-HEP-1 cell morphological changes, we compared the effect of alignment-inducing 2% PEG 600k (+0.2 μOsm/L) with 2% PEG 20,000 g mol^−1^ (PEG 20k; +10 μOsm/L) and that of 1% PEG 2M (+0.05 μOsm/L), which have a lower and higher bulk viscosity, respectively, and opposite osmotic pressure (Fig. 6A). Here, 2% PEG 600k presented a significantly higher cell anisotropy than 2% PEG 20k and 1% PEG 2M (Fig. 6B). Also, cell elongation was only observed in 2% PEG 600k (Fig. 6D), but a similar area was seen in 2% PEG 20k and 2% PEG 600k-treated cells both being significantly larger than 1% PEG 2M-treated cells (Fig. 6C) suggesting that cell area may be affected by osmotic pressure but not cell alignment and elongation. To investigate whether the volume exclusion effect induced by macromolecular crowding is the main cause of cell elongation and alignment, we used the fractional volume of occupancy (FVO) to compare the volume exclusion power of different solutions, as described previously^15^. This revealed that the 2% PEG 600k had the highest FVO (∼300% v/v) compared to 1% PEG 2M (∼259% v/v/) and 2% PEG 20k (∼28% v/v). Both 2% PEG 600k and 1% PEG 2M have a considerably higher FVO than that of the physiological extracellular microenvironment estimated to be 54% v/v^35^, but only 2% PEG 600k had an effect on elongation and alignment suggesting that FVO alone may not explain these effects. To further investigate the effects of FVO on SK-HEP-1 cell morphology we exposed these cells to the polymer polyvinylpyrrolidone 360,000 g mol^−1^ (PVP 360k) at a physiological 54% FVO (1.134% PVP 360k (w/v)) and at a supraphysiological 300% FVO (6.24% PVP 360k (w/v)) to compare the effects of these solutions with 2% PEG 600k. Elongation and increased cell anisotropy compared to 0% control were observed with both physiological and supraphysiological PVP 360k FVOs, the 6.24% PVP 360k having the strongest effect (Fig. 6E,F). However, both were significantly lower than 2% PEG 600k and neither of the PVP 360k solutions significantly enhanced cell area when compared to 0% control (Fig. 6G). Also, the bulk viscosity of 6.24% PVP 360k was comparable to that of 2% PEG 600k (Fig. 6A), suggesting the alignment effects cannot be exclusively attributed to either bulk viscosity or FVO.

### Enhanced macromolecular content increases extracellular matrix deposition

**Figure 7:**
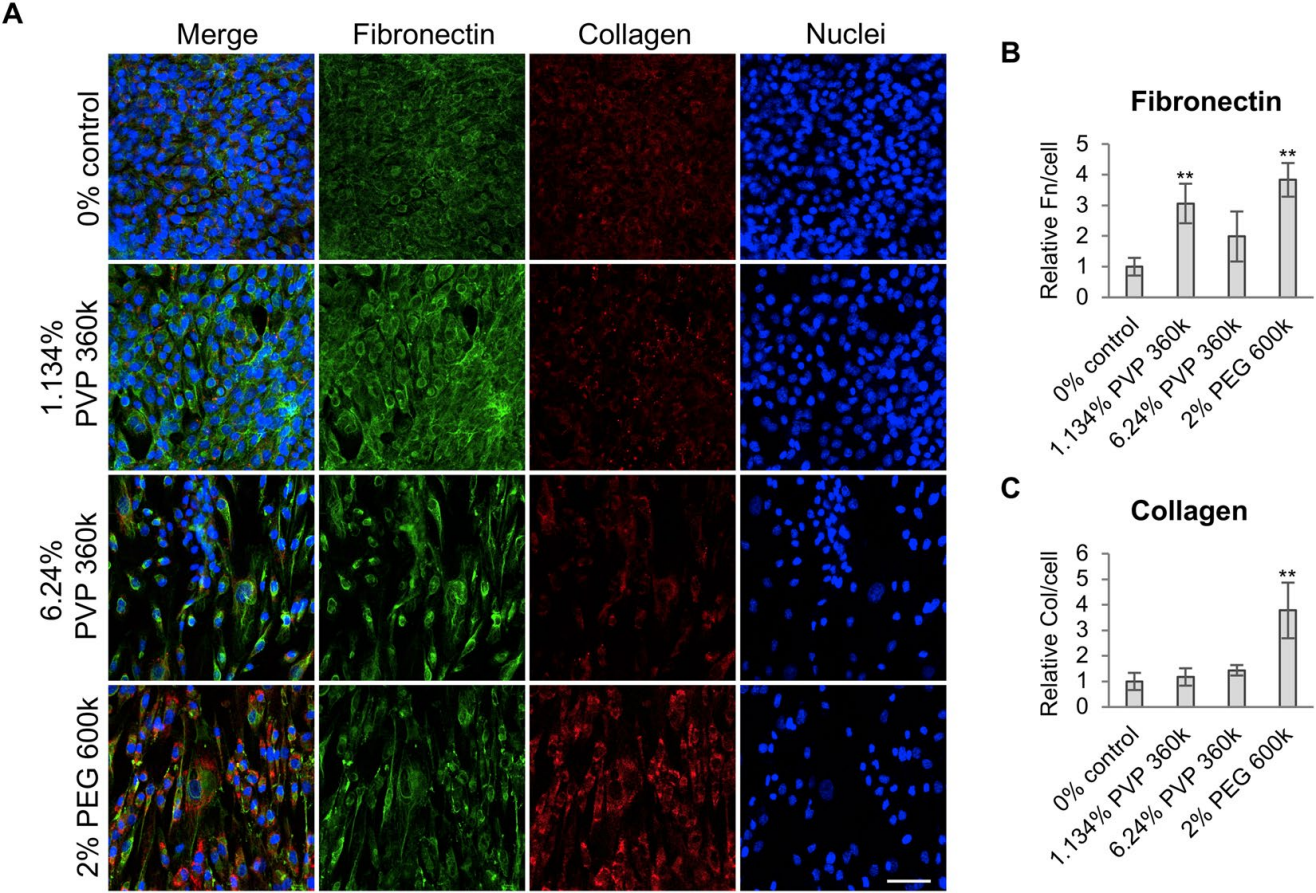
ECM deposition is enhanced differently with various polymer-containing solutions. (**A**) Immunofluorescence images of SK-HEP1 cells exposed for 4 days to polymer-containing media showing enhanced fibronectin and collagen deposition. Scale bar, 100 μm. (**B**) Quantification of fibronectin deposition from (**A**) showing enhanced deposition in polymer-treated cells. (**C**) Collagen deposition quantification from (**A**) indicating enhanced deposition in 2% PEG 600k-treated cells. Bar graphs indicate average ± S.D (n = 4).

Cellular alignment and elongation can be induced by the topographical features of the cellular substrate and the architecture of the ECM^33^. Macromolecular crowding has been shown to increase ECM deposition by human mesenchymal stem cells and fibroblasts *in vitro*^15,35^, which could explain the observed changes in SK-HEP-1 cell morphology. Hence, we investigated whether polymeric solutions would affect ECM deposition in SK-HEP-1 cells. Exposing cells to 2% PEG 600k or PVP 360k at physiological and supraphysiological FVOs enhanced the relative fibronectin deposition compared with 0% control (Fig. 7A,B), although significance was not achieved with 6.24% PVP 360k. However, only 2% PEG 600k significantly enhanced the deposition of collagen (Fig. 7A,C). These results further demonstrate that the effects observed cannot be solely the result of an enhanced FVO, and ECM deposition may not only be affected by macromolecular crowding facilitating ECM fibrillogenesis but other factors may also be at play.

### Cell spreading rate and area over compliant substrates are molecular weight-independent

**Figure 8:**
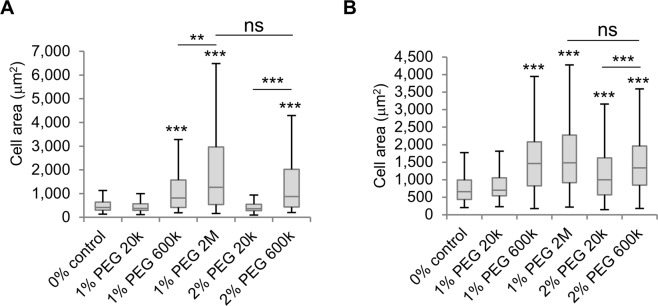
Cell spreading rate and area in cells adhered to compliant substrates correlate with bulk viscosity. (**A**) SK-HEP-1 cells attached to tissue culture polystyrene substrates and treated with PEG solutions for 1 h present differences in cell area (n ≥ 249 cells). (**B**) SK-HEP-1 cells attached to 4.5 kPa Col-PAA gels and treated with PEG solutions for 24 h present differences in cell area (n ≥ 268 cells). Boxplots represent the median, first, and third quartiles; whiskers indicate maximum and minimum within 1.5x the interquartile range. Statistical significance was assessed by one-way ANOVA with Tukey’s multiple comparison test. ns p > 0.05; **p < 0.01; ***p < 0.001.

To investigate whether the molecular weight of polymers influenced the short-term effects of macromolecules including cell spreading area of cells adhered to compliant substrates and spreading rate, solutions of PEG with varying concentration and molecular weight were used to investigate these responses. First, the area of cells spreading over a rigid polystyrene surface was analysed 1 hour after cells attached to the surface in the presence of various PEG-containing solutions to study changes in spreading rate. Cells exposed to all solutions containing PEG 600k and PEG 2M presented a significantly larger area than control cells (Fig. 8A). Interestingly, the spreading area of cells treated with comparably viscous 1% PEG 2M and 2% PEG 600k was similar, but low viscosity 2% PEG 20k did not significantly enhance the spreading rate. Likewise, cells adhered to 4.5 kPa Col-PAA gels presented differences in cell area after a 24-hour treatment with various PEG-containing solutions. Here, PEG 600k and PEG 2M also caused an enhanced cell area and no differences were observed between 2% PEG 600k and 1% PEG 2M (Fig. 8B). However, treatment with 2% PEG 20k also enhanced cell area as observed in cells adhered to polystyrene (Figs 6C and 7B), suggesting that, although the spreading rate is not affected by this solution, longer exposures induce changes in SK-HEP-1 cell area. These results demonstrate that two separate mechanisms affect cell-substrate adhesion, determined by spreading rate and area on compliant substrates, and cell alignment and elongation, as these effects occur at different timescales and in polymer-containing environments with different molecular characteristics.

## Discussion

Here, we present evidence that macromolecules of the extracellular space affect the morphology of endothelial-like cells. These effects were observed in cancer-derived SK-HEP-1 cells with endothelial origin but not in non-cancerous HUVECs (Figs 1 and 3) indicating that this response is not shared by all endothelial cell types. In the context of the TME, the data presented here suggest that cancer-derived endothelial cells might respond to high-macromolecular-content environments such as those found in mucinous tumours, which have been reported to promote angiogenesis and vessel invasion^36,37^. However, the morphological changes observed in SK-HEP-1 cells, which also present mesenchymal features, do not necessarily correspond to those observed in normal endothelial cells but could be closer to mesenchymal cells such as fibroblasts or mesenchymal stromal cells, which typically have an elongated spindle-like morphology and form partially aligned monolayers in two-dimensional cultures. In the TME, mesenchymal cells such as cancer-associated fibroblasts facilitate cancer progression and endothelial cells have been proposed as a source of cancer-associated fibroblasts through the process known as endothelial-to-mesenchymal transition^38^. Thus, SK-HEP-1 cells exposed to pathological-like macromolecular contents could result in cell alignment due to an endothelial-to-mesenchymal transition. Hence, an extensive phenotypical characterisation of cells exposed to high-macromolecule-content environments could then shed light on the mechanisms behind these observations.

Studying the molecular players of macromolecule-induced effects will also contribute to the general understanding of the biological role of microenvironmental soluble macromolecules. The actin cytoskeleton and related proteins such as various Rho GTPases are involved in sensing and transmitting mechanical signals in cells^39^. However, here we show that the effects induced by high macromolecular content on cell alignment, although altered by the pharmacological inhibition of the Rho GTPases Rac1 and cdc42, are independent of actin cytoskeleton integrity (Fig. 3E and Supplementary Fig. S2). Other cellular molecules that could be affected by these microenvironments include various mechanosensitive ion channels which have been previously shown to respond to viscosity and cause endothelial cell alignment^40–43^. The sole expression of these ion channels does not explain the differences observed between HUVECs and SK-HEP-1 cells as these ion channels are also expressed in HUVECs^43,44^. Besides cellular structures, the ECM has a central role in determining cell morphology and phenotype. As previously reported^15^, we show that polymer-containing solutions increased fibronectin and collagen deposition (Fig. 7). The reconstitution of ECM under macromolecular crowding conditions has been shown to enhance the formation of tubular structures by HUVECs^45^. Moreover, the content and nano-architecture of the ECM have been demonstrated to affect cell morphology and alignment^46,47^ indicating that the increased ECM deposition observed here could induce the morphological changes experienced by SK-HEP-1 cells. However, whether the observed changes in ECM are required and sufficient to induce SK-HEP-1 cell elongation and alignment remains unclear.

To define better the characteristics of macromolecular microenvironments leading to cellular morphological changes and cell alignment, we used different polymer-containing solutions with varying FVO, bulk viscosity, molecular weight, concentration, and polymer type. The resulting data show that cellular alignment and elongation are highly dependent on the *molecular weight* of the macromolecules used rather than the bulk viscosity of the solution and indicate that high FVO tends to correlate with these cellular characteristics (Figs 5 and 6). Conversely, in the short-term effect of high-macromolecular-content solutions on the formation of cell-substrate bonds, demonstrated by increased cell spreading rate and total cell area over compliant substrates, the dominating factor was found to be the *bulk viscosity* of the solution (Fig. 8). A recent study has shown how volume exclusion alone is not responsible for all the effects caused by microenvironmental macromolecules on collagen deposition but factors such as negative charge and polydispersity, which is higher in solutions containing low molecular weight polymers, must be taken into account to understand the effect of macromolecular crowding^48^. In line with this report, PVP 360k and PEG 600k with equal FVO and comparable bulk viscosity did not induce the same degree of morphological changes, cellular alignment, and ECM deposition, suggesting that other characteristics of these microenvironments also play a role in influencing these effects. Furthermore, the different effects obtained with PEG molecules of low and high molecular weight may also be due to the higher nano-viscosity (viscosity at the nanoscale) of low molecular weight molecules compared to high molecular weight ones even if the macro-viscosity (bulk viscosity) of the solution is equal. If the diameter of the molecule(s) affected by the presence of polymers in the solution is considerably smaller than the radius of gyration of the polymer, the nano-viscosity experienced by the molecule will be considerably lower than the macro-viscosity of the solution, but if the radius of gyration of the polymer is smaller than the diameter of the affected molecule, it will experience the macro-viscosity of the solution^49–51^. Thus, the nano-viscosity experienced by molecules exposed to 1% PEG 2M will be lower than the nano-viscosity experienced in 2% PEG 600k depending on their size.

## Conclusion

In conclusion, the results presented here suggest that incorporating polymers of physiologically or pathologically relevant macromolecular size, concentration, and viscosity into the extracellular microenvironment greatly influences cell behaviour. These changes include both bulk viscosity-induced effects on cellular substrate sensing and bulk viscosity-independent effects on cell elongation, alignment, and ECM deposition (Supplementary Fig. S3). Also, this work indicates that the role of macromolecular crowding and viscosity within the TME should be studied further and blocking the secretion of soluble macromolecules such as mucins by cancer cells could be considered as a therapeutic strategy in cancer as an alternative to ECM normalisation therapies^52^.

## Materials and Methods

### Cell culture

SK-HEP-1 cells (ATTC) were maintained in Minimum Essential Medium with alpha modification (MEMalpha, GE Healthcare) supplemented with 10% (v/v) foetal calf serum (FCS, Hyclone) and 100 IU/ml penicillin and 0.1 mg/ml streptomycin (Gibco), and 1.25 μg/ml fungizone (Gibco). SK-HEP-1 cells were always used at passages lower than passage 20. Human umbilical vein endothelial cells (HUVEC, Promocell) were maintained in endothelial cell growth medium (Promocell) supplemented with endothelial cell growth medium supplement mix (Promocell). HUVECs were used at passages lower than passage 9. Cell media were replaced every 2–3 days. Before reaching confluency, cells were harvested or split using TrypLE select (Gibco).

### Polymer-containing media preparation

Polymeric solutions were prepared as 2X concentrated solutions. High viscosity G-rich Na-alginate (FMC Biopolymer), dextran from *Leuconostoc* spp. M_w_ 450,000–650,000 (Sigma), and polyethylene glycol (PEG) M_w_ 20,000, 600,000, 1,000,000, or 2,000,000 (Sigma) and polyvinylpyrrolidone (PVP) M_w_ 360,000 (Sigma) were dissolved in MilliQ water containing 0.15 M NaCl and 15 mM HEPES (Gibco) adjusted at pH 7.4. 1X solutions were prepared by mixing 50% of 2X polymer solutions and 50% complete medium. For controls, 50% complete medium was mixed with MilliQ water containing 0.15 M NaCl and 15 mM HEPES adjusted at pH 7.4. Solutions were filter-sterilised prior to exposure to cells, and were not kept for more than 4 weeks. Viscosities of freshly prepared 1X solutions were measured with a cone/plate viscometer (Brookfield DV-II + Pro) with a CP(E)-41 spindle at shear rates ranging from 2 to 300 s^−1^ at a controlled temperature of 37 °C. Each solution was measured at least 3 times. All viscosity values in the main text correspond to values at 100 s^−1^ shear rate.

### Fluorescence microscopy

Medium was removed and cells were immediately fixed with cold 4% paraformaldehyde in Dulbecco’s Phosphate Buffered Saline (DPBS) for 15 min. After fixation, cells were washed twice with DPBS and permeabilised with 0.1% Triton X-100 for 5 minutes. Next, samples were blocked for 1 h with 3% Bovine Serum Albumin in DPBS. For actin cytoskeleton staining, phalloidin-488 (1:1000) (SCBT) was used and incubated for 1 h in the dark. Finally, nuclei were stained with Hoechst 33342 for 2 min at room temperature in the dark, and samples were washed extensively with DPBS. Images were acquired with an INCell Analyser 2200 automated microscope with a CMOS camera. To analyse ECM deposition, cells were fixed with cold 100% methanol for 15 min, washed and blocked as described above. Samples were stained with primary antibodies against human collagens I/II/III/IV/V (1:50, Bio-rad) and fibronectin (1:200,Sigma) overnight at 4 °C in the dark. Cells were washed and incubated with PE (1:500, Santa Cruz Biotechnology) and Alexa Fluor-488 (1:500, Life Technologies) secondary antibodies, respectively, for 1 h at room temperature in the dark. Nuclei were stained as before, and images were captured with Nikon Eclipse Ti-E microscope with Hamamatsu Flash 4.0 sCMOS camera and Nikon C2 Confocal with PMTs for 3 channel simultaneous imaging.

### Polyacrylamide gel preparation

To prepare polyacrylamide gels, glass coverslips were washed twice with 70% ethanol, activated with 0.1 M NaOH, and functionalised by silanizing with (3-aminopropyl)trimethoxysilane (Sigma). Subsequently glass coverslips were treated with 0.5% glutaraldehyde (Sigma) for 30 min and washed extensively with MilliQ water. Mixtures of MilliQ water, acrylamide monomers (Sigma), and crosslinker *N*,*N* methylene-bis-acrylamide (Sigma) were prepared according to previously determined formulations^53,54^. To start the polymerisation reaction, 5 μl of 10% ammonium persulphate (Sigma) and 0.75 μl *N*,*N*,*N*′,*N*′-tetramethylethylenediamine (TEMED, Sigma) were added into 0.5 ml mixtures. To allow cell adhesion, gels were treated with 1 mg/ml *N*-sulphosuccinimidyl-6-(4′-azido-2′-nitrophenylamino) hexanoate (sulpho-SANPAH, Sigma) which was activated by UV light. Finally, gels were incubated with 10 μg/ml collagen I from rat tail overnight at 4 °C and UV-sterilised prior to cell seeding.

### Cell morphology analysis

Phase contrast images were taken from live or fixed cells with a Nikon TE200 microscope equipped with a Nikon DS-Fi1c camera and DS-U2 PC control unit. NIS Elements software was used to define cell periphery manually and obtain the total cell area and circularity (4π × area/perimeter^2^).

### Cell proliferation

For cell proliferation, 5 × 10^4^ cells per well were seeded in 12-well plates and allowed to attach overnight. After a 4-day treatment, cells were harvested and pelleted by centrifugation. Total nuclei were counted by automated cell nuclei quantitation following the manufacturer’s specifications (Nucleocounter).

### Cell alignment and orientation analysis

Phase-contrast images of cell monolayers were taken at X10 magnification with a Nikon TE200 microscope with a Nikon DS-Fi1c camera and DS-U2 PC control unit and the NIS-Element Microscope Imaging software. Pseudo-coloured images were obtained with the OrientationJ plugin^55,56^ of ImageJ using a Gaussian window of 2 pixels, a Fourier gradient, and colours representing orientation (hue) and coherency (saturation). Fibre anisotropy was calculated using the Fibriltool macro^57^ by analysing 5 square (200 × 200 pixels) regions of interest per image and 4 images per condition in each experiment.

### Inhibition studies

Pharmacological inhibition was conducted using 2 μg/ml cytochalasin B (Sigma) for actin filament disruption, 20 μM blebbistatin (Medchem) for myosin II inhibition, 20 μM PF-573228 (Medchem), 20 mM Y-27632 (Medchem) as a ROCK inhibitor, 50 μM NSC23566 as a selective inhibitor of Rac1, (Medchem) or 50 μM ML-141 for the selective inhibition of cdc42 (Medchem) in polymer-containing or control media on SK-HEP-1 cells for 4 days.

### Fractional volume of occupancy studies

The fractional volume occupancy (FVO) was calculated as previously described^36^ using values for hydrodynamic radii or obtained by the best-fit trend line applied to the data^54^. To determine fractional volume of occupancy effect and subsequent, extracellular matrix deposition, cells were seeded at 12500 per well in a 24-well plate and left to attach overnight. Next, medium was removed, and cells treated with 1.134% and 6.24% PVP 360k and 2% PEG 600k prepared in medium containing 100 μM L-ascorbic acid (Sigma). Controls were also cultured in medium supplemented with 100 μM L-ascorbic acid. Cells were treated in triplicate for 4 days and imaged at the end of that period.

### Statistical analyses

Statistical analyses were carried out using GraphPad Prism or Microsoft Excel softwares. When two groups were compared, two-tailed Student’s t-test was used, and when more groups were compared, analysis of variance (ANOVA) was carried out. For data not meeting normal distribution, equivalent non-parametric tests were applied. Significance was considered at p < 0.05. Details of specific tests, repeats, sample numbers, and data and error descriptions are specified in figure captions.

## Supplementary information

Supplementary figures

## Data Availability

The datasets generated during and/or analysed during the current study are available from the corresponding author on reasonable request.
