# Supplementary material for: The extracellular fluid macromolecular composition differentially affects cell-substrate adhesion and cell morphology
Source: Sci Rep. 2019 Jun 11;9:8505. doi: 10.1038/s41598-019-44960-3 (PMC6560040; doi:10.1038/s41598-019-44960-3)
Supplement: Supplementary file 1 — Supplementary figures [file 41598_2019_44960_MOESM1_ESM.pdf]

# Supplementary Information

## The extracellular macromolecular composition differentially affects cell-substrate adhesion and cell morphology

Jordi Gonzalez-Molina[1,2,3]*, Joana Mendonça da Silva[1], Barry Fuller[4], Clare Selden[1].

[1] UCL Institute for Liver and Digestive Health, UCL - Royal Free Hospital Campus, UCL Medical School, NW3 2PF, London, UK.

[2] Microbiology, Tumor, and Cell biology Department. Karolinska Institutet, Stockholm, Sweden.

[3] Oncology-Pathology Department, Karolinska Instituet, Stockholm, Sweden.

[4] Department of Surgical Biotechnology, Royal Free Hospital, UCL Medical School, NW3 2QG, London, UK.

*Correspondence to: j.molina@ucl.ac.uk

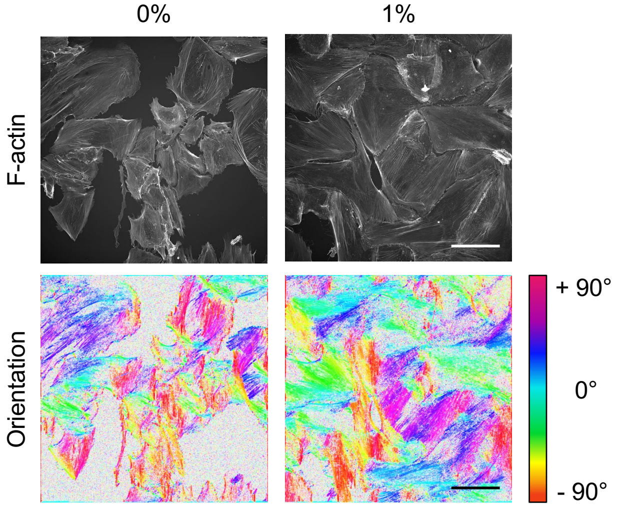


**Supplementary Fig. S1. Na-alginate does not cause cell alignment in HUVECs.** Fluorescence images and orientation of actin fibres in HUVECs exposed to control (0%) or 1% Na-alginate-containing (1%) medium for 4 days. Scale bar, 50 μm.

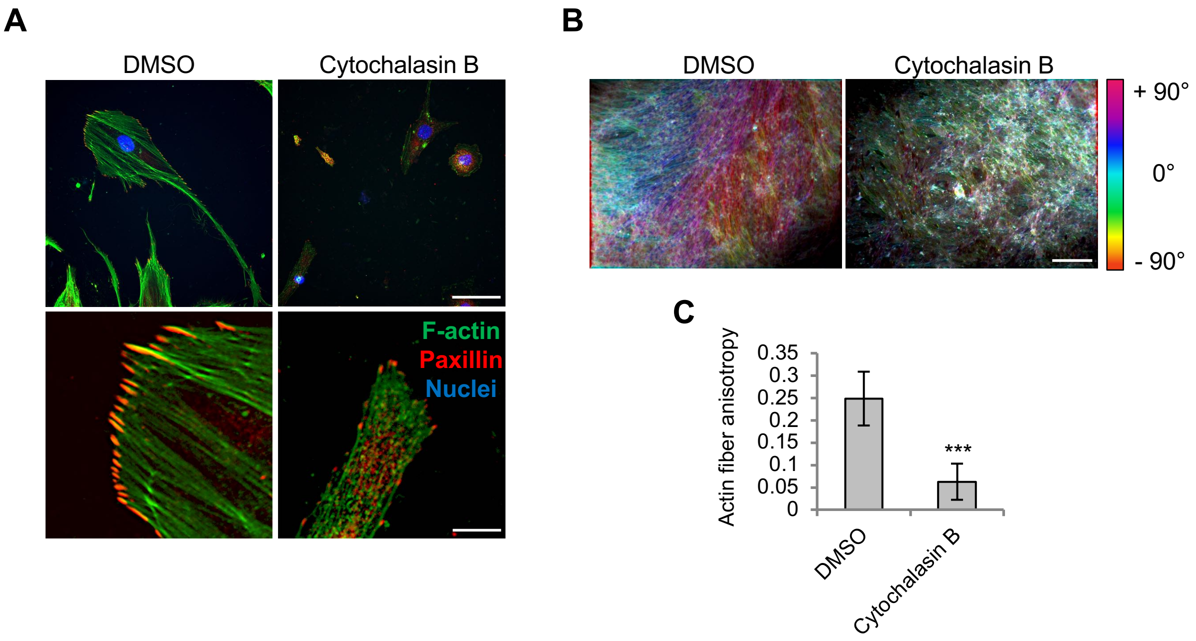


**Supplementary Fig. S2. Cytochalasin B effectively disrupts the actin cytoskeleton in 1% Na-alginate-containing medium. A**, Fluorescence images of the actin cytoskeleton and paxillin-containing focal adhesions of cells exposed to 1% Na-alginate control (DMSO) or actin-disrupted (Cytochalasin B) medium for 4 days. Scale bars, 25 μm (top images) and 5 μm (bottom images). **B**, Orientation of actin fibres of of SK-HEP-1 cells monolayers exposed to control (DMSO) or actin-disrupting (Cytochalasin B) 1% Na-alginate-containing medium for 4 days. Scale bar, 100 μm. **C**, quantification of actin fibre anisotropy of cells from (**B**) (n = 60 fields in 3 independent experiments). Bars represent the average ± s.d. Statistical significance was assessed by Student's *t*-test. *** $p < 0.001$.

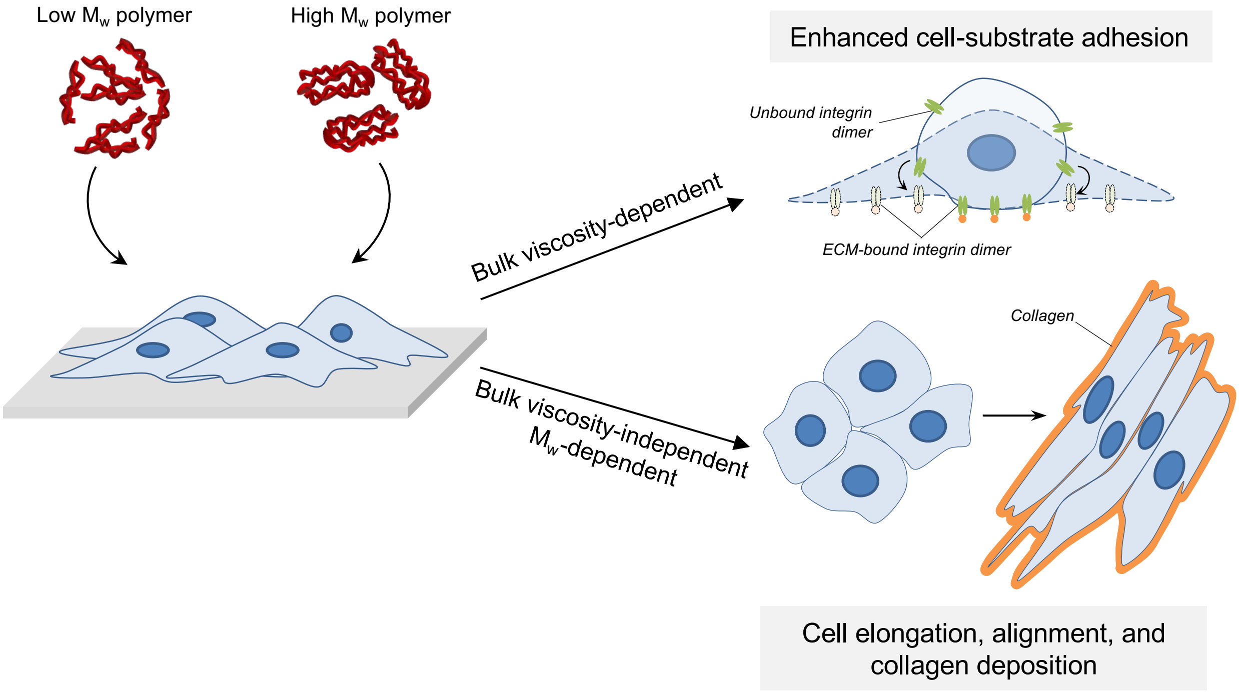


**Supplementary Fig. S3. Graphical summary of the effect of soluble polymers on SK-HEP-1 cells.** The introduction of polymers into the cell growth media has various effects on SK-HEP-1 cells depending on the characteristics of these polymers. Cell-substrate adhesion is facilitated by polymers in bulk solution viscosity-dependent manner. However, morphological changes including cell elongation and multicellular alignment and the increased deposition of collagen are independent of the bulk viscosity of the solution but appears to be a polymer molecular weight-dependent phenomenon.
